# Supplementary figures and images for: CRISPR targeting of FOXL2 c.402C>G mutation reduces malignant phenotype in granulosa tumor cells and identifies anti‐tumoral compounds
Source: Mol Oncol. 2025 Jan 8;19(4):1092–116. doi: 10.1002/1878-0261.13799 (PMC11977662; doi:10.1002/1878-0261.13799)

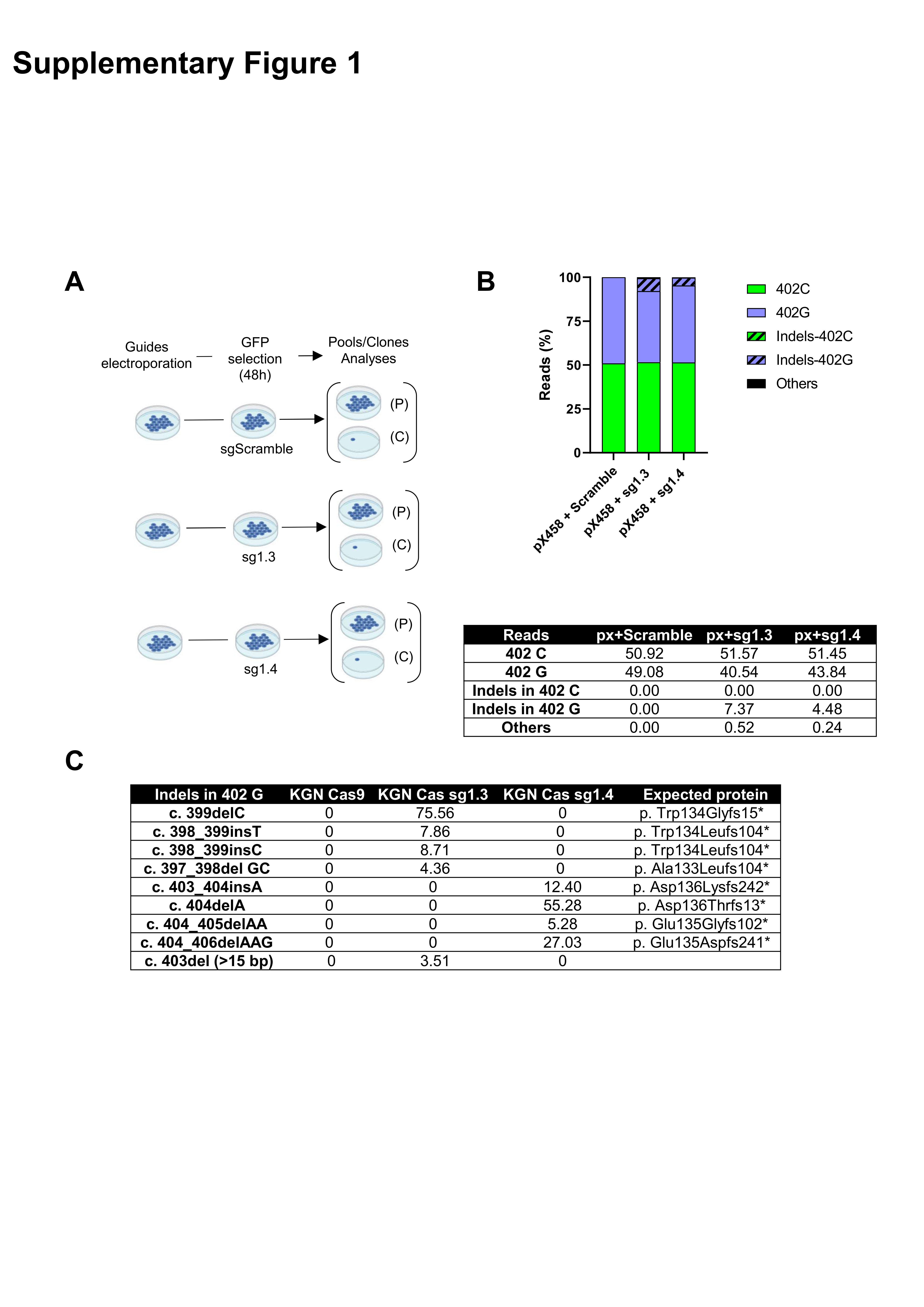

Supplement: Supplementary file 1 — Fig. S1. Edition of FOXL2 c.402C>G mutation in KGN cells upon transfection of CRISPR/Cas9 complexes. (A) Timeline for the generation of pools (P) and clones (C) after the electroporation of CRISPR/Cas9 complexes. (B) Analysis of the specificity of the guide RNAs for the FOXL2 c.402C>G mutant allele. Graph represents percentage of reads matching each edited and not edited alleles for the pool of nucleofected cells for each condition. The bottom table shows the numerical data of the graph. (C) Indels identified in nuleofected pools, upon activity of each CRISPR/Cas9 complex. [file MOL2-19-1092-s002.jpg]

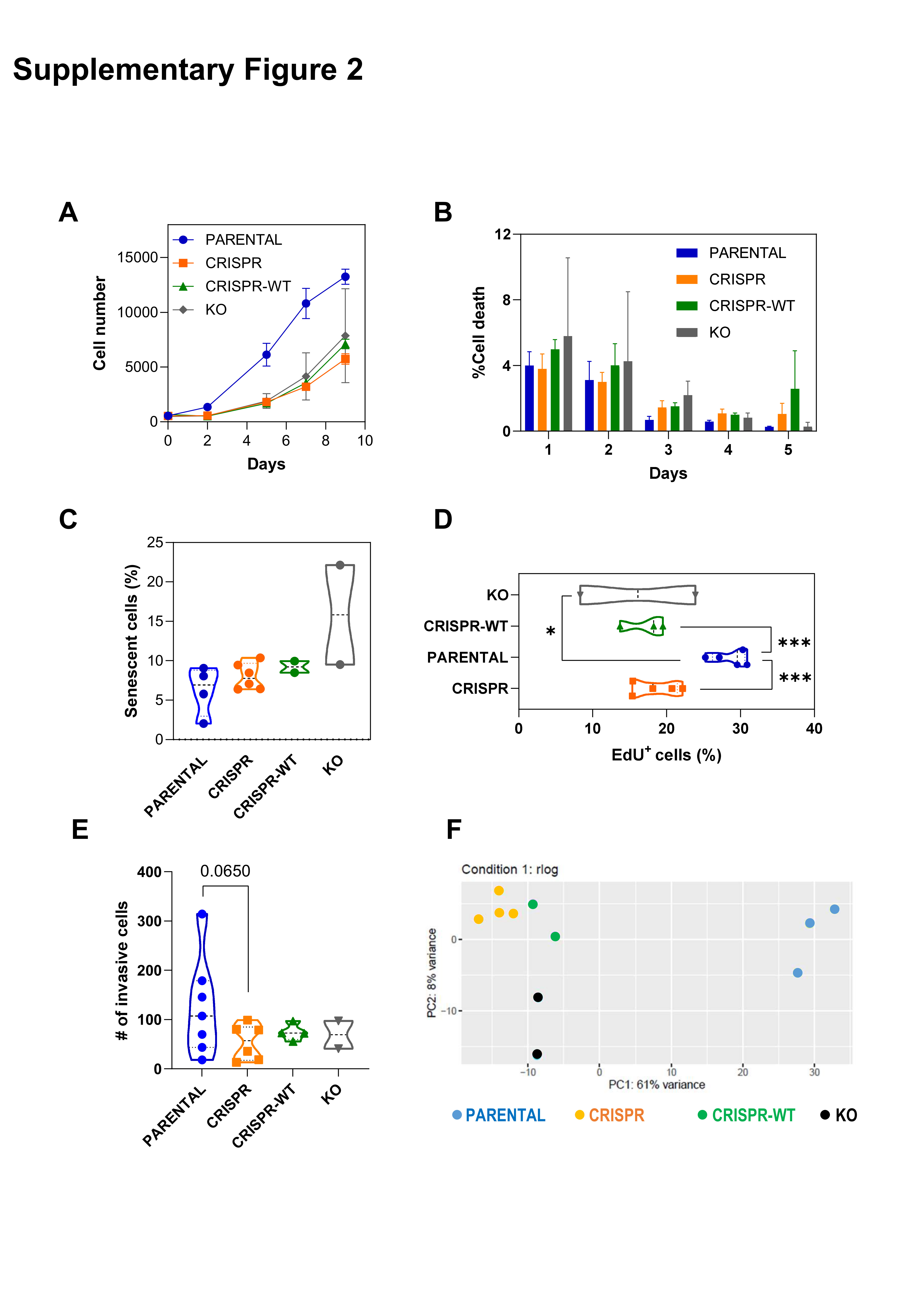

Supplement: Supplementary file 2 — Fig. S2. Characterization of null clones and CRISPR‐edited clones in which only wild type allele can be detected. Panels A–D present data in Figure 2(A–D), separating CRISPR‐edited clones and clones with only the WT allele, and including results from the two FOXL2‐null clones isolated in this study. Panels E and F similarly separate clone types to display data from Figures 4A and 5A, respectively. In most assays, KO null cells and WT‐only clones behave similarly to CRISPR clones; however, principal component analysis (PCA) of transcriptomic data reveals a distinct expression signature for the two KO clones compared to both CRISPR‐edited and parental lines. [file MOL2-19-1092-s018.jpg]

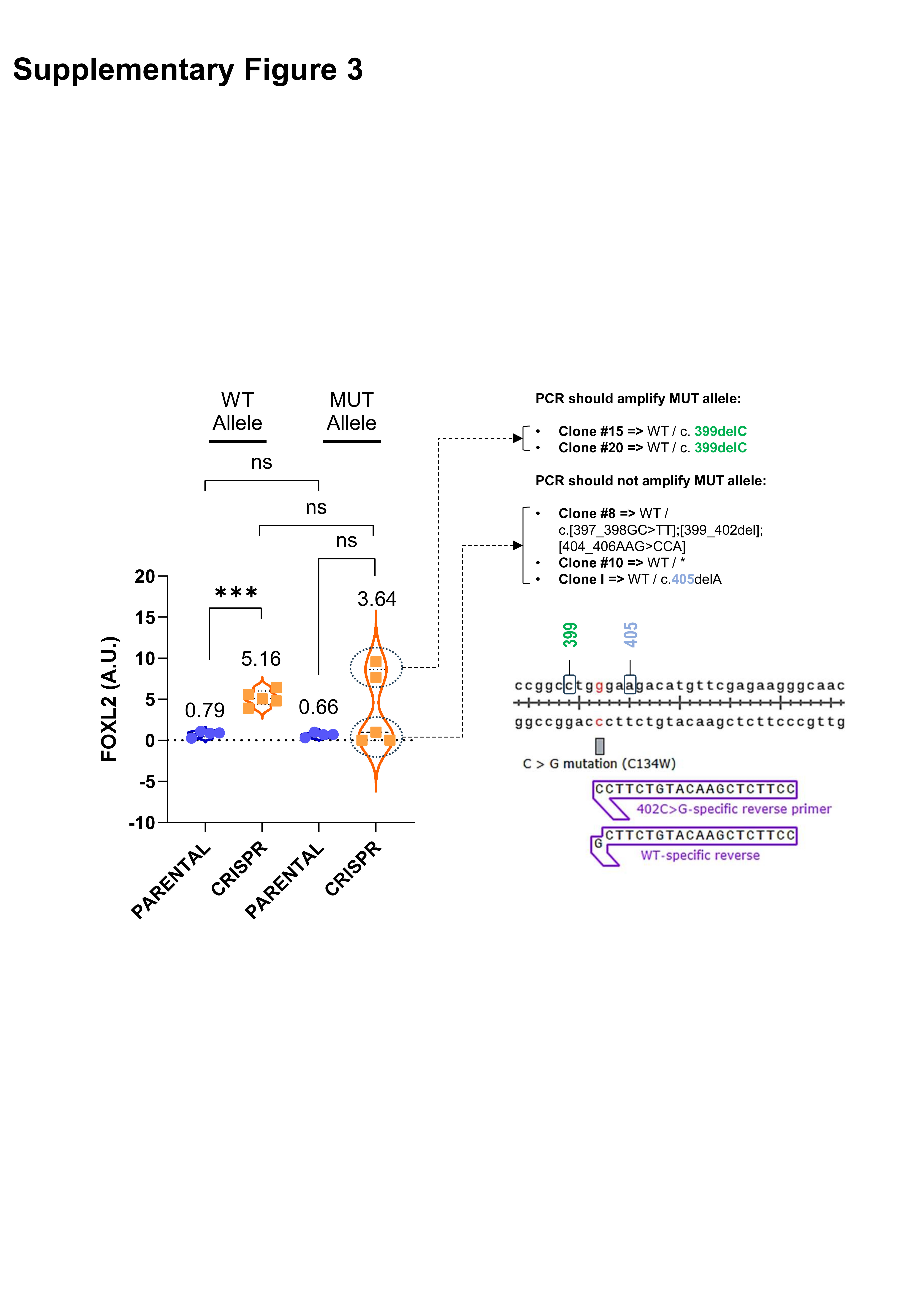

Supplement: Supplementary file 3 — Fig. S3. Overexpression of FOXL2‐WT and FOXL2‐mutant alleles in KGN cells depleted from C134W mutation. Using primer pairs as described by Shin et al. [34], we selectively amplified and quantified FOXL2‐WT and FOXL2‐MUT alleles in both PARENTAL (blue, n = 4) and CRISPR (orange, n = 5) clones. Average expression values are shown. The right panel provides details on the status of the MUT allele in the CRISPR clones used, along with specific reverse primer locations and edited nucleotide positions. A significant overexpression of the WT allele is observed in CRISPR clones compared to PARENTAL cells. Even greater overexpression is detected for the MUT allele in CRISPR clones where the MUT allele remains amplifiable (#15 and #20). [file MOL2-19-1092-s012.jpg]

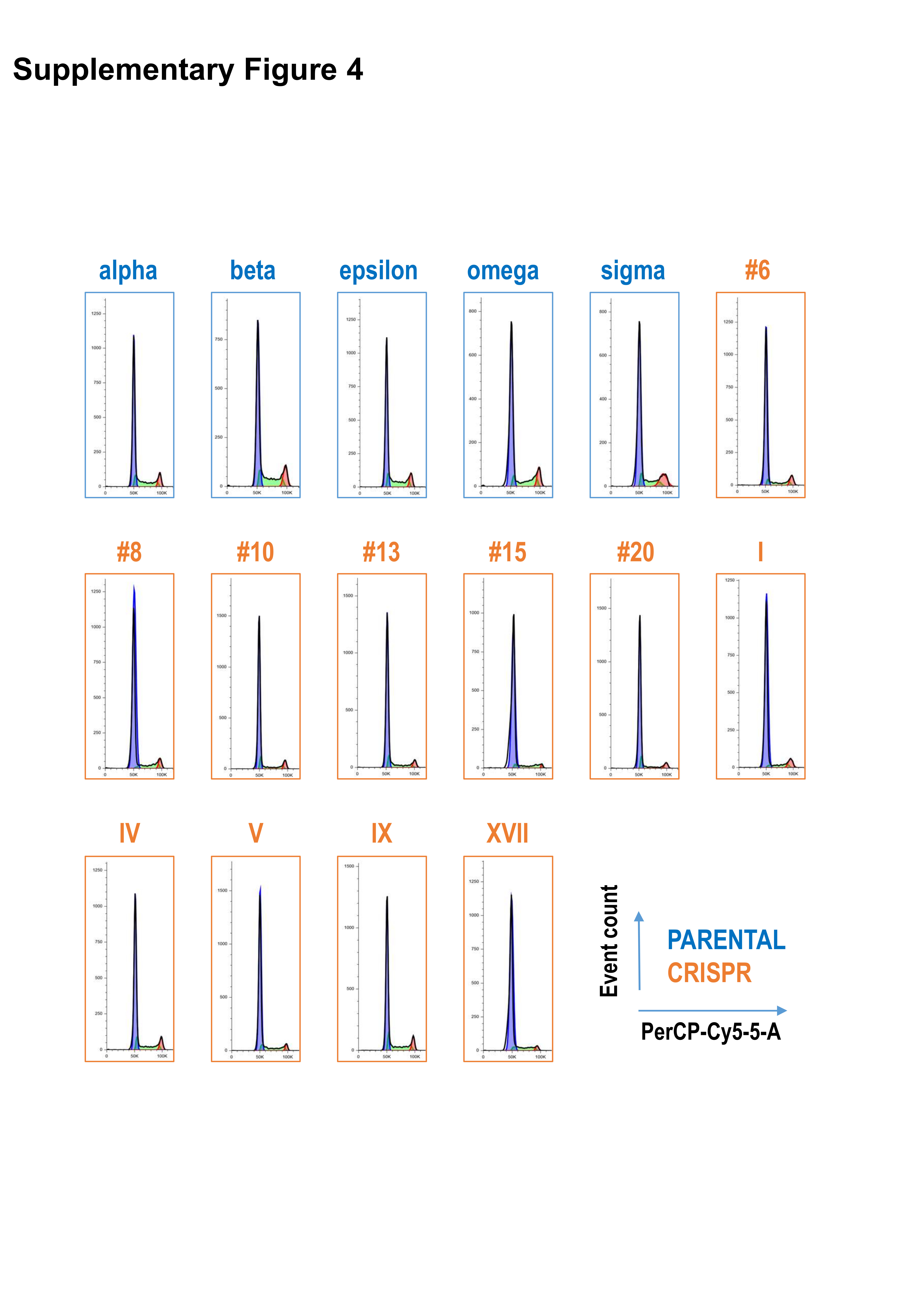

Supplement: Supplementary file 4 — Fig. S4. Flow cytometry cell cycle analysis of PARENTAL and CRISPR clones. Cell cycling profiling was determined by PI‐staining and flow cytometry analysis. Data were analyzed with Floreada.io (https://floreada.io/analysis). Graphs show cell cycle profiles of PARENTAL (n = 5) and CRISPR (n = 11) clones. [file MOL2-19-1092-s015.jpg]

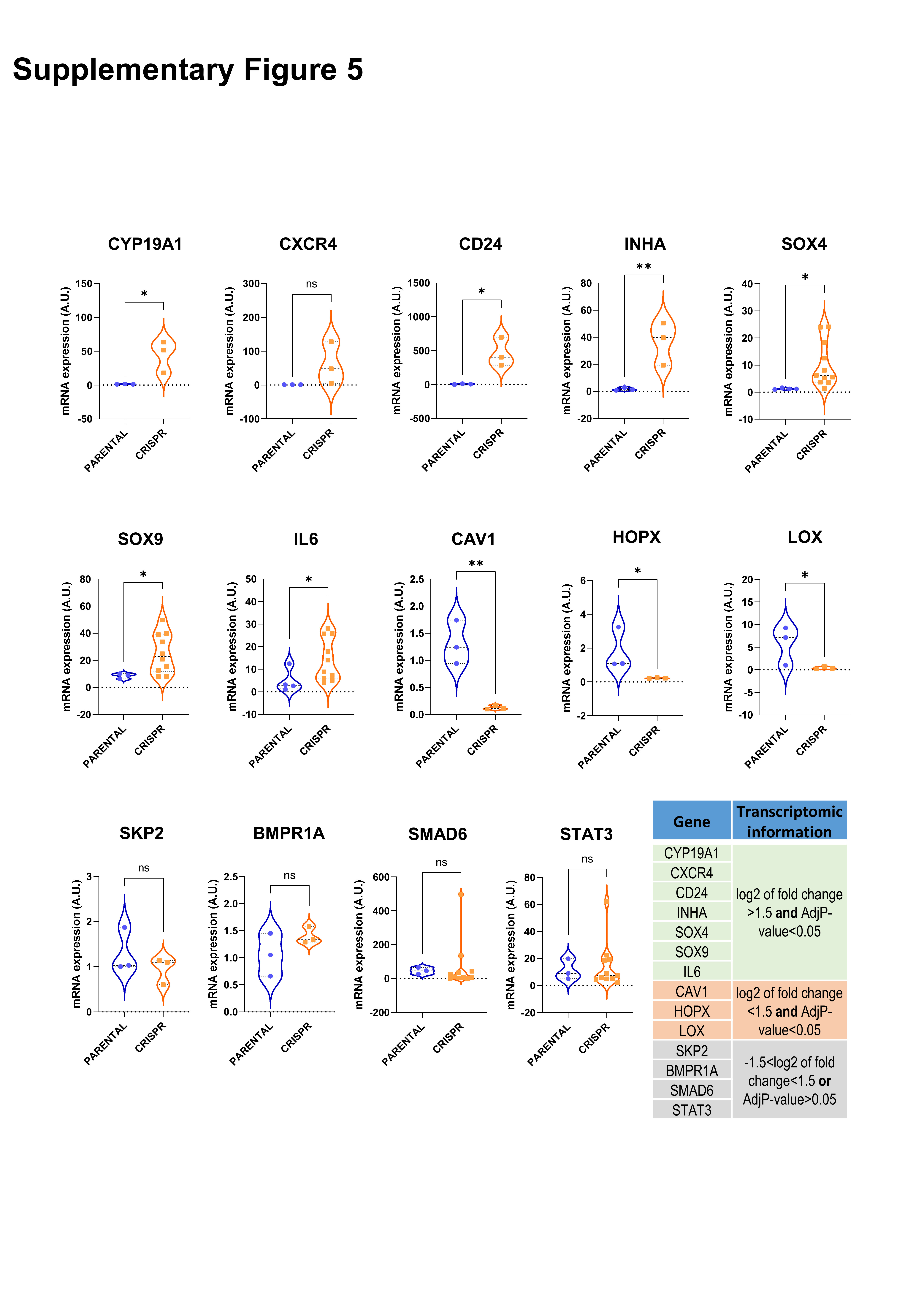

Supplement: Supplementary file 5 — Fig. S5. Validation by RT‐qPCR of transcriptomic data. Graphs show the results from quantitative PCR results of genes included in the transcriptomic study. Transcriptomic information from the RNAseq experiment (Figure 5) is included in the bottom right table. [file MOL2-19-1092-s006.jpg]

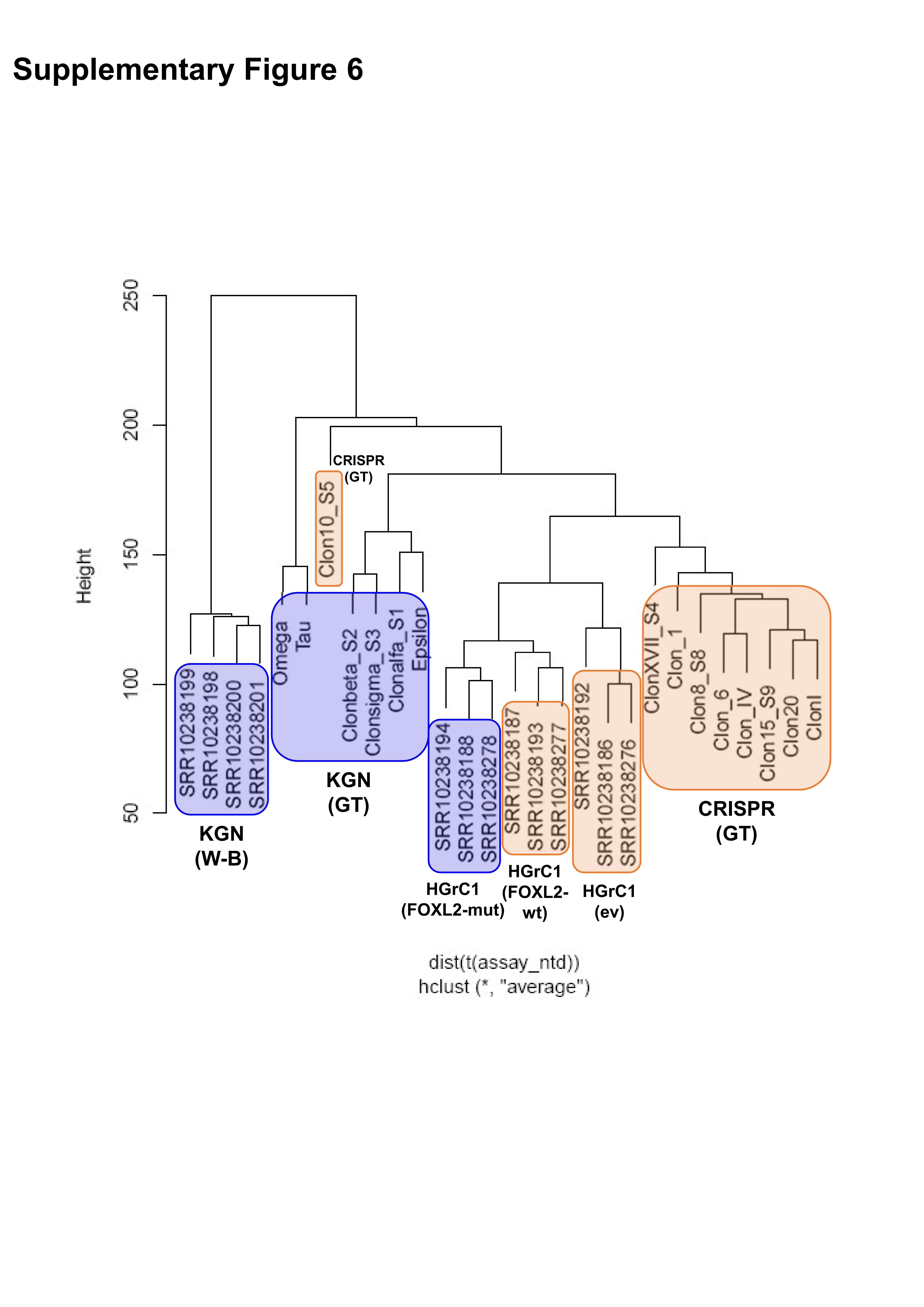

Supplement: Supplementary file 6 — Fig. S6. Clustering analyses of transcriptomic data from tumoral and non‐tumoral granulosa cell clones. PARENTAL [KGN(GT)] and CRISPR [CRISPR(GT)] clones from this study are compared with KGN and HGrC1 granulosa cell lines generated by Weis‐Banke et al [26]: [KGN (W‐B)], [HGrC1 (ev)], [HGrC1 (FOXL2‐mut)] and [HGrC1 (FOXL2‐wt)]. KGN/PARENTAL genotype cells are marked in blue, and CRISPR/Edited/WT cells are colored in orange. [file MOL2-19-1092-s011.jpg]

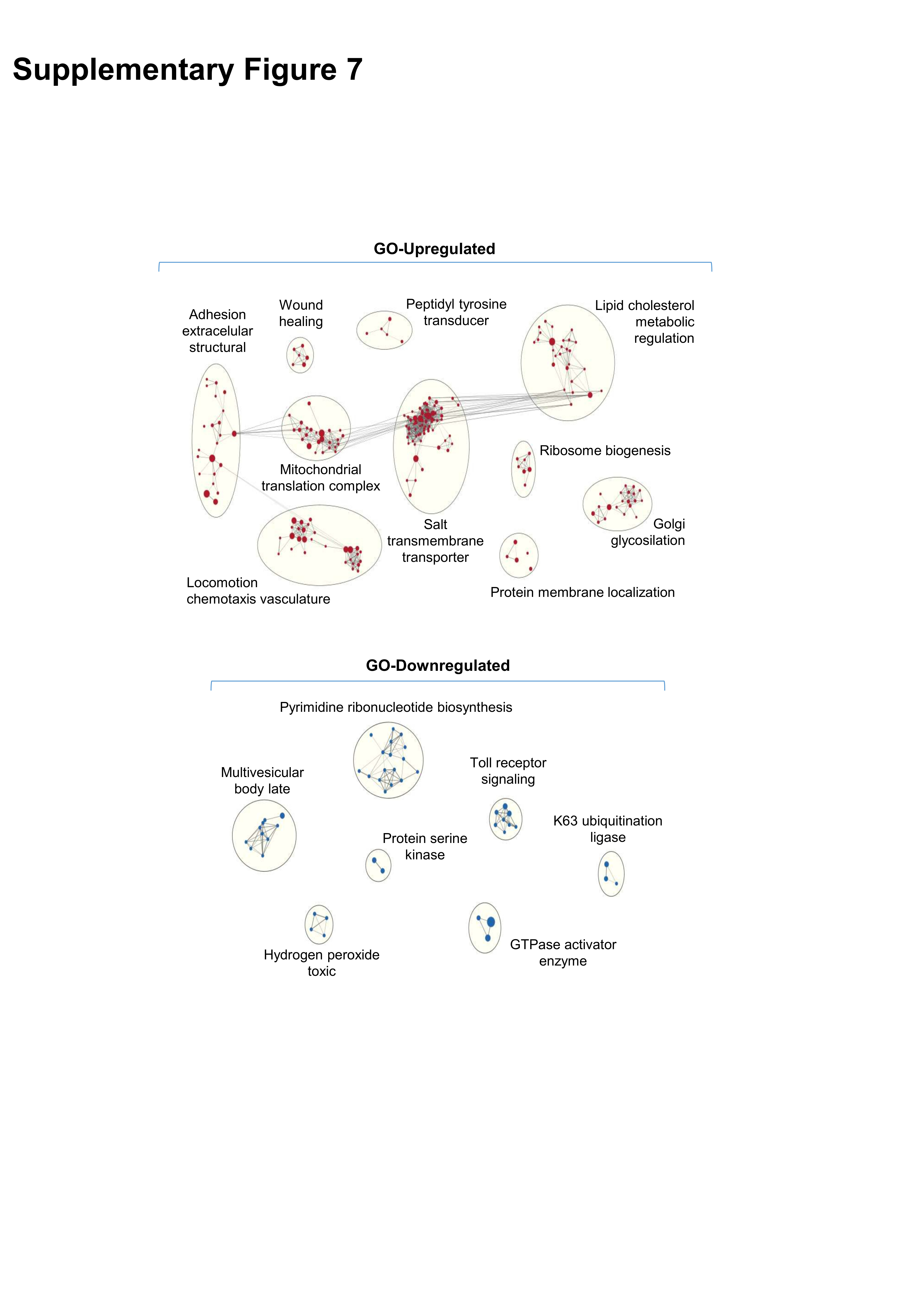

Supplement: Supplementary file 7 — Fig. S7. Network clustering of proteomic GO terms from differential expressed proteins. Up and down‐regulated, GO terms at q‐value < 0.05 are shown. [file MOL2-19-1092-s016.jpg]

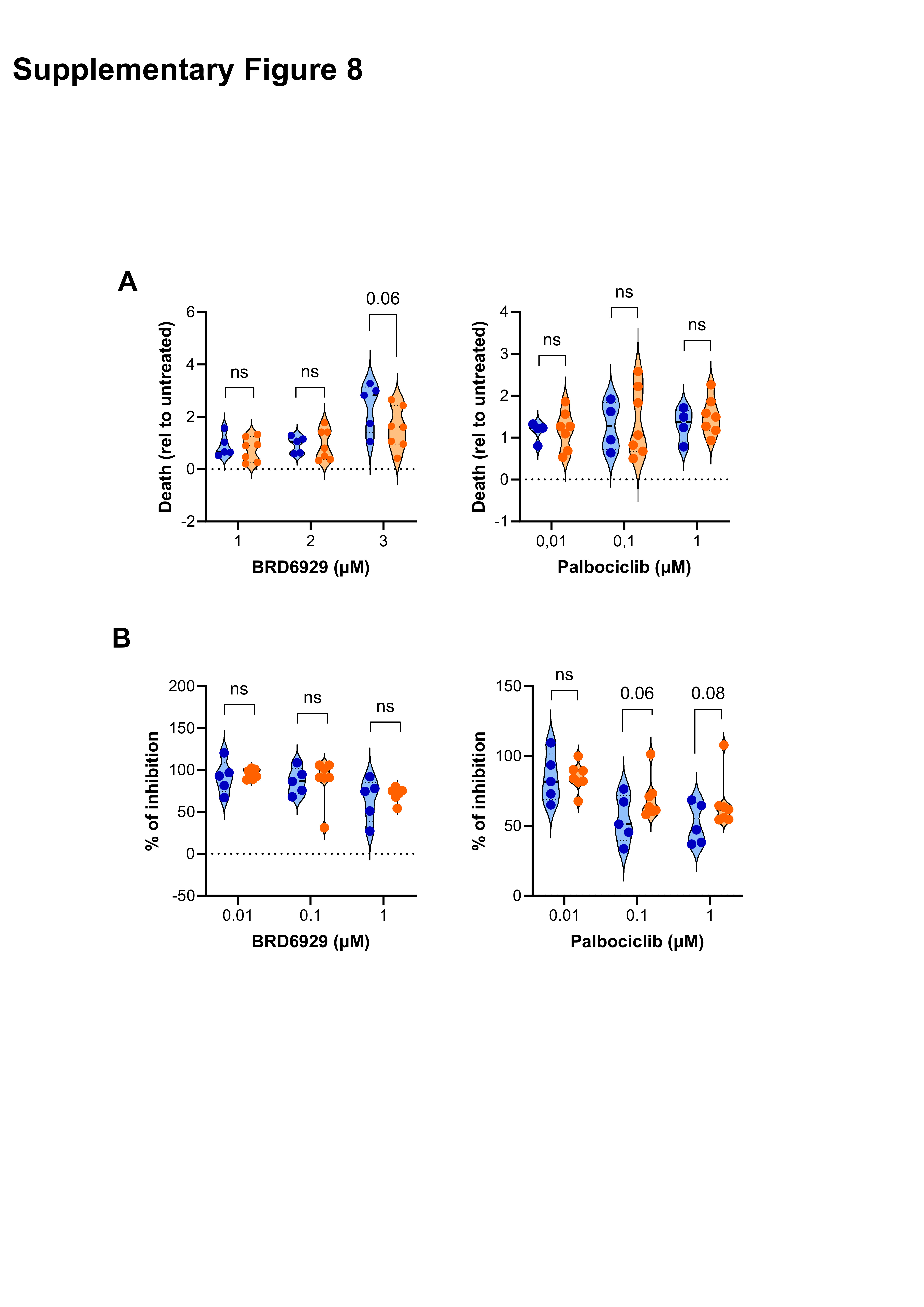

Supplement: Supplementary file 8 — Fig. S8. Comparison of the effects induced by Palbociclib and BRD6929 in PARENTAL and CRISPR clones. Quantification of cell death (A) and cell number (B) is shown for both PARENTAL (n = 5) and CRISPR (n = 6) clones, 24 h after treatment with palbociclib or BRD6929. Values are normalized to those of untreated cells. Near‐significant comparisons are indicated with exact P values. [file MOL2-19-1092-s013.jpg]
