# Supplementary material for: CRISPR targeting of FOXL2 c.402C>G mutation reduces malignant phenotype in granulosa tumor cells and identifies anti‐tumoral compounds
Source: Mol Oncol. 2025 Jan 8;19(4):1092–116. doi: 10.1002/1878-0261.13799 (PMC11977662; doi:10.1002/1878-0261.13799)
Supplement: Supplementary file 9 — Table S1. Genomic analysis from pools after gene edition. [file MOL2-19-1092-s014.pdf]

Supplementary Table 1. Genomic analysis from pools after gene edition. Table includes percentage of reads for the wild-type allele (402C), mutant allele (402G), total editions on mutant (Indels in 402G) and wild-type (Indels in 402C) alleles and, finally, for edited reads in which allele origin cannot be determined (Others).

Allele	%		
	**KGN Cas9**	**KGN Cas sg1.3**	**KGN Cas sg1.4**
**402 C**	50,21	61,24	73,85
**402 G**	49,57	3,44	8,83
**Indels in 402 C**	0,00	0,00	0,00
**Indels in 402 G**	0,00	25,04	14,31
**Others**	0,22	10,27	3,02
