# Supplementary material for: CRISPR targeting of FOXL2 c.402C>G mutation reduces malignant phenotype in granulosa tumor cells and identifies anti‐tumoral compounds
Source: Mol Oncol. 2025 Jan 8;19(4):1092–116. doi: 10.1002/1878-0261.13799 (PMC11977662; doi:10.1002/1878-0261.13799)
Supplement: Supplementary file 10 — Table S2. Indels characterization of the reads obtained from amplicon Deep sequencing of the pools after gene edition. [file MOL2-19-1092-s005.pdf]

Supplementary Table 2. Indels characterization of the reads obtained from amplicon Deep sequencing of the pools after gene edition.

Indels in 402 G	%			Expected protein
	KGN Cas9	KGN Cas sg1.3	KGN Cas sg1.4	
c. **399delC**	0	65,81	1,76	p. Trp134Glyfs15*
c. **392_402del**	0	10,60	0	p. Asp131Glyfs103*
c. **398_399insT**	0	7,64	0	p. Trp134Leufs104*
c. **398_399insC**	0	3,56	0	p. Trp134Leufs104*
c. **399_400delCT**	0	2,57	0	p. Trp134Glyfs103*
c. **398_399insA**	0	2,49	0	p. Trp134Leufs104*
c. **397_398del GC**	0	2,06	0	p. Ala133Leufs104*
c. **398_399insG**	0	1,84	0	p. Trp134Leufs104*
c. **395_399del**	0	1,33	0	p. Pro132Leufs104*
c. **400delT**	0	1,07	0	p. Trp134Glyfs15*
c. **399_401delCTG**	0	1,03	0	p. Trp134Glufs142*
**c. 403_404insA**	0	0	46,21	p. Asp136Lysfs242*
**c. 404delA**	0	0	26,04	p. Asp136Thrfs13*
**c. 404_405delAA**	0	0	9,79	p. Glu135Glyfs102*
**c. 404_406delAAG**	0	0	6,20	p. Glu135Aspfs241*
**c. 403_404insAA**	0	0	5,91	p. Asp136Lysfs242*
**c. 403_404insC**	0	0	2,26	p. Glu135Alafs103*
**c. 404_407del**	0	0	1,83	p. Glu135Alafs13*
