# Supplementary material for: CRISPR targeting of FOXL2 c.402C>G mutation reduces malignant phenotype in granulosa tumor cells and identifies anti‐tumoral compounds
Source: Mol Oncol. 2025 Jan 8;19(4):1092–116. doi: 10.1002/1878-0261.13799 (PMC11977662; doi:10.1002/1878-0261.13799)
Supplement: Supplementary file 11 — Table S3. List of clones generated from pools edited with guides sg1.3 and sg1.4. [file MOL2-19-1092-s008.pdf]

**Supplementary Table 3. List of clones generated from pools edited with guides sg1.3 and sg1.4.**

| ID | TYPE | Indel at allele | Genotype | Protein (expected for edited allele) |
|---|---|---|---|---|
| **K_E1_sg13_3pulso** | CRISPR | 402G | **WT / c.398_399insT** | p. Trp134Leufs104* |
| **K_E1_sg13_1** | CRISPR | 402G | **WT / c. 399delC** | p. Trp134Glyfs15* |
| **K_E1_sg13_6** | CRISPR | 402G | **WT / c. 399delC** | p. Trp134Glyfs15* |
| **K_E1_sg13_8** | CRISPR | **Other** | **WT / c.[397_398GC>TT];[399_402del]; [404_406AAG>CCA]** | p. Ala133Leufs15* |
| **K_E1_sg13_11** | CRISPR | 402G | **WT / c. 399delC** | p. Trp134Glyfs15* |
| **K_E1_sg13_12** | CRISPR | 402G | **WT / c. 389_398del** | p. Asp131Glyfs103* |
| **K_E1_sg13_13** | CRISPR | 402G | **WT / c.398_399insT** | p. Trp134Leufs104* |
| **K_E1_sg13_15** | CRISPR | 402G | **WT / c. 399delC** | p. Trp134Glyfs15* |
| **K_E1_sg13_17** | CRISPR | 402G | **WT / c. [399_400insT]; [398C>T]** | p. Trp134Leufs104* |
| **K_E1_sg13_18** | CRISPR | 402G | **WT / c. 389_398del** | p. Asp131Glyfs103* |
| **K_E1_sg13_20** | CRISPR | 402G | **WT / c. 399delC** | p. Trp134Glyfs15* |
| **K_E1_sg14_IIpulso** | CRISPR | 402G | **WT / c.403delG** | p. Glu135Lysfs14* |
| **K_E1_sg14_I** | CRISPR | 402G | **WT / c.405delA** | p. Asp136Thrfs14* |
| **K_E1_sg14_III** | CRISPR | 402G | **WT / c.406_407insAA** | p. Asp136Glufs14* |
| **K_E1_sg14_IV** | CRISPR | **Other** | **WT / c.396_403del** | p. Ala133Argfs102* |
| **K_E1_sg14_VII** | CRISPR | 402G | **WT / c. 403_404insA** | p. Asp136Argfs102* |
| **K_E1_sg14_XIV** | CRISPR | 402G | **WT / c. 403_404insA** | p. Asp136Argfs102* |
| **K_E1_sg14_XVI** | CRISPR | 402G | **WT / c. 403_404insA** | p. Asp136Argfs102* |
| **K_E1_sg14_XVIII** | CRISPR | 402G | **WT / c.406_407insAA** | p. Asp136Glufs14* |
| **K_E1_sg14_XX** | CRISPR | **Other** | **WT / c. 402_404del** | p. *134 |
| **K_E1_sg14_VI** | CRISPR | 402G | **WT / c.396_403del** | p. Ala133Argfs102* |
| | | | | |
| **K_E1_sg14_IIIp** | KO | 402C | **c. 404delA** | p. Asp136Thrfs13* |
| **K_E1_sg14_XXI** | KO | 402C | **c. 403_404insA** | p. Asp136Argfs102* |
| | | | | |
| **K_E1_sg14_IX** | CRISPR | 402G | **WT / *** | p. 376* |
| **K_E1_sg13_10** | CRISPR | 402G | **WT / *** | p. 376* |
| **K_E1_sg14_V** | CRISPR | 402G | **WT / *** | p. 376* |
| **K_E1_sg14_XVII** | CRISPR | 402G | **WT / *** | p. 376* |
