# Supplementary material for: CRISPR targeting of FOXL2 c.402C>G mutation reduces malignant phenotype in granulosa tumor cells and identifies anti‐tumoral compounds
Source: Mol Oncol. 2025 Jan 8;19(4):1092–116. doi: 10.1002/1878-0261.13799 (PMC11977662; doi:10.1002/1878-0261.13799)
Supplement: Supplementary file 13 — Table S5. List of top25 up (red) and top25 down (blue) regulated genes differentially expressed between PARENTAL and CRISPR clones. [file MOL2-19-1092-s004.pdf]

Supplementary Table 5. List of top25 up (red) and top25 down (blue) regulated genes differentially expressed between PARENTAL and CRISPR clones.

GENE	Function and relationship with cancer	Relation with granulosa cells and/or FOXL2
**AOX1** Aldehyde oxidase 1	It produces hydrogen peroxide and it is implicated in ROS. It has been related to different cancer types including pancreatic cancer (PDAC), clear cell renal cell carcinoma (ccRCC) and colorectal cancer (CRC).	Enriched in heat-stressed granulosa cells (doi: 10.3390/biology11060839)
**IFRD1** Interferon Related Developmental Regulator 1	Protein related to interferon gamma. Transcriptional co-activator/repressor that controls the growth and differentiation of specific cell types during embryonic development and tissue regeneration. Protein expression in colon cancer is associated with poorer patient prognosis.	Ifrd1 mRNA is primarily induced in granulosa cells during the periovulatory period in the rat ovary (doi: 10.1002/MRD.22673).
**CAV1** Caveolin 1	Main component of the caveolae plasma membranes. Negative regulator of RAS signaling. Links integrin to Ras-ERK signaling. It has been related to various cancer types, including head and neck squamous cell carcinoma, melanoma, and Ewing's sarcoma family tumors.	It plays a role in folliculogenesis and female reproduction (doi: 10.1093/HUMREP/DEY299). In bovine ovaries, CAV1 is expressed in granulosa and theca cells of the follicle and large and small cells of the corpus luteum (doi: 10.1002/MRD.20513).
**TBC1D9** TBC1 Domain Family Member 9	Activation of GTPase activity and intracellular protein transport Associated with the inhibition of migratory and invasive capabilities of colorectal cancer cells. Downregulated in breast cancer.	None
**CAV2** Caveolin 2	Major component of the inner surface of caveolae, small invaginations of the plasma membrane, and is involved in essential cellular functions, including signal transduction, lipid metabolism, cellular growth control and apoptosis CAV2 plays a significant role in cancer progression and metastasis in various types of cancer, including head and neck squamous cell carcinoma (HNSCC), breast cancer, and pancreatic cancer.	None
**SSX2IP** SSX Family Member 2 Interacting Protein	It is involved in the formation of the actin cytoskeleton at adherens junctions. It is also a centrosome maturation and maintenance factor. Associated with different cancer types including nasopharyngeal carcinoma, acute lymphoblastic leukemia, hepatocellular carcinoma and myeloid leukemia.	None

**ACTA2**<br>Actin Alpha 2, Smooth Muscle	Implicated in actin polymerization and nucleotide binding. Six actin different proteins.<br>High expression of ACTA2 is associated with a worse overall survival and response to immune checkpoint inhibitors in gastric cancer. Also related to relapse in glioma patients, being involved in a higher migration and malignant phenotype.	Upregulated in theca interna when compared with granulosa cells (doi: 10.1371/journal.pone.0119800)
**TMEM178B**<br>Transmembrane Protein 178B	This transmembrane protein is a negative regulator of inflammatory cytokine production.<br>A TMEM178B-BRAF fusion has been reported in a malignant melanoma.	None
**RAB3B**<br>RAB3B, Member RAS Oncogene Family	Enables GDP binding activity; GTPase activity; and myosin V binding activity. Involved in several processes, including positive regulation of dopamine uptake involved in synaptic transmission; regulation of synaptic vesicle cycle; and regulation of vesicle size.<br>Up-regulated in different cancer types including gastric cancer, hepatoma cells and gliomas.	None
**MYRF**<br>Myelin Regulatory Factor	Transcription factor involved in olygodendrocyte differentiation, and regulator of Cardiac and Early Gonadal Development.<br>Related to pancreatic cancer progression and testicular germ cell tumors.	None
**LOX**<br>ysyl Oxidase	Tumor suppresor via canonical and non-canonical TGFB signaling. It promotes the crosslinking between ECM collagen type I, collagen type III and elastin.<br>Involved in multple cancers.	It plays a critical role in the regulation of granulosa cell differentiation being involved in tissue remodeling and ECM (doi: 10.1530/REP-16-0254)
**VSNL1**<br>Visinin Like 1	Neuronal calcium sensor protein. Directly or indirectly regulates the activity of adenylyl cyclase.<br>Associated with cholangiocarcinoma, colorectal carcinogenesis, glioblastoma and squamous cell carcinoma.	None
**DNAH11**<br>Dynein Axonemal Heavy Chain 11	Microtubule-dependent motor ATPase involved in the movement of respiratory cilia.<br>It has been connected to colon cancer, pituitary adenomas, ovarian and breast cancer.	None
**AXL**<br>AXL Receptor Tyrosine Kinase	Tyr-K receptor. Involved in several cellular functions including growth, migration, aggregation and anti-inflammation in multiple cell types.<br>Associated with different tumor types.	Involved in the viability of hypoxic granulosa cells (doi: 10.1002/jcp.31162). Expressed by granulosa cells at high levels throughout antral follicle development.

**CARMN**<br>Cardiac Mesoderm Enhancer-Associated Non-Coding RNA	Predicted to be involved in regulation of gene expression.<br>Associated with multiple tumor types.	None
**CSPG4**<br>Chondroitin Sulfate Proteoglycan 4	CSPG4 can mediate intracellular signalling downstream of growth factor receptor and integrin interactions, potentiating communication between the extracellular and intracellular compartments of the cell.<br>Related to several tumor types.	Upregulated in TGFβ-induced GCTs, when compared with wild type granulosa cells (doi: 10.3390/cancers14092184)
**TAGLN**<br>Transgelin	It is involved in calcium-independent smooth muscle contraction. It acts as a tumor suppressor, and the loss of its expression is an early event in cell transformation and the development of some tumors, coinciding with cellular plasticity.	Upregulated in TGFβ-induced GCTs when compared with wild type granulosa cells (doi: 10.3390/cancers14092184).
**LCP1**<br>Lymphocyte Cytosolic Protein 1	Actin-binding protein. It plays a role in the activation of T-cells in response to costimulation through TCR/CD3 and CD2 or CD28 and modulates the cell surface expression of IL2RA/CD25 and CD69.<br>Associated with cancer.	None
**CD274**<br>PD-L1	PDL-1. Inhibitory receptor ligand that is expressed by hematopoietic and non-hematopoietic cells, such as T cells and B cells and various types of tumor cells.<br>Key immune checkpoint player considered to be prognostic in many types of human malignancies, including colon cancer and renal cell carcinoma.	Not expressed in granulosa cell baseline tumors (doi: 10.1007/s10637-020-01043-9)
**JPH2**<br>Junctophilin 2	Component of junctional complexes that mediate cross talk between cell surface and intracellular ion channels.<br>Abnormally expressed in leimyosarcoma and spcifically methylated in gastric cancer.	Upregulated in TGFβ-induced GCTs when compared with wild type granulosa cells (doi: 10.3390/cancers14092184).
**FGF1**<br>Fibroblast Growth Factor 1	Fibroblast growth factor 1. Mitogenic and cell survival activities<br>Correlates with poor survival in various cancers and resistance to platinum-based chemotherapy of serous cancers.	Favors survival of COV434 granulosa tumor cells upon etoposide treatment (doi: 10.1038/s41389-018-0033-y).<br>Involved in the differentiation of ovarian follicles.
**FBN2**<br>Fibrillin 2	Component of connective tissue microfibrils that may be involved in elastic fiber assembly.<br>Abnormal methylation/expression in diferent tumor types.	None
**TRPC4**<br>Transient Receptor Potential Cation Channel Subfamily C Member 4	It forms a non-selective calcium-permeable cation channel that is activated by Gq-coupled receptors and tyrosine kinases and plays a role in multiple processes including endothelial permeability, vasodilation, neurotransmitter release and cell proliferation.<br>Associated with prognosis, tumor microenvironment and treatment sensitivity.	None

**SLC8A1**<br>Solute Carrier Family 8 Member A1	Low affinity, high capacitance calcium antiporter membrane protein that functions to regulate intracellular calcium concentrations.<br>Related to Megacolon and Penile cancer.	Target gene of FOXL2 (PANTHER CLASS as: cell communication and signal transduction) (doi: 10.7554/eLife.04207)
**LMOD1**<br>Leiomodin-1	Required for proper contractility of visceral smooth muscle cells, it mediates nucleation of actin filaments.<br>Deregulated in endometrial cancer (doi: 10.1038/s41598-020-66872-3).	Involved in ovulation in mouse ovary (doi: 10.1101/2023.08.21.554210).
**SMIM3**<br>Small integral membrane protein 3	Transmembrane protein involved in cell channel regulation and associated with neuronal differentiation.<br>Related to pheochromocytonas, oral squamous cell carcinomas and acute myeloid leukemia.	None
**KSR1**<br>Kinase suppresor of Ras1	Enables 14-3-3 protein binding activity, ATP binding activity, and protein C-terminus binding activity. Involved in positive regulation of MAPK cascade.<br>Associated with different tumor types.	None
**AHRR**<br>Aryl Hydrocarbon Receptor Repressor	It participates in the aryl hydrocarbon receptor (AhR) signaling cascade, which mediates dioxin toxicity, and is involved in regulation of cell growth and differentiation.<br>Related to cancer at different levels.	Activated in granulosa cells upon exposure to contaminants (doi: 10.1016/j.tox.2017.07.003)
**HTR7**<br>5-Hydroxytryptamine Receptor 7	Serotonin receptor.<br>Abnormally regulated in breast, gastric, laryngeal cancers and acute myeloid leukemia.	None
**LIMCH1**<br>LIM And Calponin Homology Domains 1	Enables myosin II head/neck binding activity. Involved in several processes, including cytoplasmic actin-based contraction involved in cell motility; positive regulation of stress fiber assembly; and regulation of focal adhesion assembly.<br>Related with some cancer types.	None
**ARL4C**<br>ADP Ribosylation Factor Like GTPase 4C	Member of the ADP-ribosylation factor family of GTP-binding proteins.<br>Related with cancer processes.	None
**GPRC5C**<br>G Protein-Coupled Receptor Class C Group 5 Member C	It may mediate the cellular effects of retinoic acid on the G protein signal transduction cascade.<br>Some roles reported in cancer.	Upregulated in KGN treated with forskolin (doi: 10.1210/en.2004-0889)
**UST**<br>Uronyl 2-Sulfotransferase	It transfers sulfate to the 2-position of uronyl residues, such as iduronyl residues in dermatan sulfate and glucuronyl residues in chondroitin sulfate.<br>It may play a role in cancer progression and could potentially serve as a biomarker for predicting patient survival in certain types of cancer.	None

**ABCC4**<br>ATP Binding Cassette Subfamily C Member 4	It transports various molecules across extra- and intra-cellular membranes.<br>It is involved in muti-drug resistance.<br>It plays an important role in cancer progression and may serve as a potential therapeutic target in certain types of cancer.	Mediator of ovulation in granulosa cells. Involved in prostaglandin signaling (doi: 10.1096/fj.202101931RR)
**CDH12**<br>Cadherin 12	A type II classical cadherin of the cadherin superfamily. It mediates calcium-dependent cell-cell adhesion and appears to be expressed specifically in the brain.<br>It appears to be involved in cancer progression and may serve as a potential therapeutic target in these malignancies.	Granulosa cell marker (doi:10.3389/fendo.2019.00832)
**CYP11A1**<br>Cytochrome P450 Family 11 Subfamily A Member 1	Member of cytochrome P450 superfamily of enzymes, which catalyze many reactions involved in drug metabolism and synthesis of cholesterol, steroids and other lipids. This protein localizes to the mitochondrial inner membrane and catalyzes the conversion of cholesterol to pregnenolone, the first and rate-limiting step in the synthesis of the steroid hormones.<br>It has been implicated in different cancers (breast, kidney, renal, squamous cell and skin).	It plays a crucial role in steroid hormone synthesis in granulosa cells. Increase expression in GCs undergoing luteinization in ovulation (doi: 10.1210/en.2016-1264. Epub 2016 Jul 18). No expression in granulosa cell tumors (doi: 10.1158/0008-5472.CAN-05-1024). FOXL2 represses the activity of the mouse Cyp11a1 promoter (doi: 10.1530/REP-11-0259).
**STC2**<br>Stanniocalcin 2	Secreted, homodimeric glycoprotein that is expressed in a wide variety of tissues and may have autocrine or paracrine functions.<br>Upregulated in various cancers, it is a biomarker for many of them.	Involved in the regulation of apoptosis and autophagy in granulosa cells (doi: 10.1016/j.yexcr.2023.113473)
**TSKU**<br>Tsukushi, Small Leucine Rich Proteoglycan	It enables transforming growth factor beta binding activity. Involved in organ development and cholesterol efflux and homeostasis.<br>TSKU expression is associated with poor overall survival in non-small cell lung cancer.	None
**GNG2**<br>G Protein Subunit Gamma 2	One of the gamma subunits of a guanine nucleotide-binding protein. Involved in signaling mechanisms across membranes.<br>It acts as a tumor suppresor in breast cancers (doi: 10.1038/s41419-022-04690-3). Low expression levels in malignant melanomas.	Upregulated in granulosa cells from primary follicles (doi:10.3389/fcell.2018.00085)
**COL4A5**<br>Collagen Type IV Alpha 5 Chain	One of the six subunits of type IV collagen, the major structural component of basement membrane.<br>Related to the progression of lung and breast cancer.	None
**CSGALNACT1**<br>Chondroitin Sulfate N-Acetylgalactosaminyltransferase 1	It transfers N-acetylglucosamine (GalNAc) to the core tetrasaccharide linker and to elongating chondroitin sulfate chains in proteoglycans.<br>Few works associate this gene with cancer.	Involved in maturation of follicular granulosa cells in buffalo (doi:10.1186/s12864-018-5208-6)

**NTNG2**<br>Netrin G2	Predicted to be involved in several processes, including basement membrane assembly; cell morphogenesis involved in differentiation; and regulation of cell projection organization.<br>Moderate association with different cancer types.	None
**NCK2**<br>NCK Adaptor Protein 2	Member of NCK family of adatpor proteins. Bind and recruits various proteins involved in regulation of receptor protein tyr-k.<br>Few reports associate this gene with cancer.	None
**SLC14A1**<br>Solute Carrier Family 14 Member 1	Membrane transporter that mediates urea transport in erythrocytes.<br>Biomarker in some cancers. Associated with the progression of different tumors.	None
**JCAD**<br>Junctional Cadherin 5 Associated	Endothelial cell to cell junction protein.<br>It plays a role in the development and progression of certain types of cancer.	None
**CD55**<br>CD55 Molecule	Glycoprotein involved in the regulation of the complement cascade.<br>Weakly associated with cancer.	Recently identified as a cell surface marker that can be used to isolate early-stage granulosa cells (doi: 10.1111/cpr.13589)
**TMOD1**<br>Tropomodulin 1	Actin-capping protein that regulates tropomyosin by binding to its N-terminus, inhibiting depolymerization and elongation of the pointed end of actin filaments and thereby influencing the structure of the erythrocyte membrane skeleton.<br>It may play a role in promoting cancer cell growth and could be a potential target for therapeutic interventions.	Involved in Regulation of Ovarian Granulosa Cell Morphogenesis, Development and Differentiation (doi: 10.3390/ijms20163966)
**CALB2**<br>Calbindin 2	Intracellular calcium-binding protein belonging to the troponin C superfamily. This protein plays a role in diverse cellular functions, including message targeting and intracellular calcium buffering.<br>It plays a role in cancer progression and may serve as a potential biomarker or therapeutic target in certain cancer types.	Useful marker for GCTs (doi: 10.1309/GRH4-JWX6-J9J7-QQTA)
**AC022075.1**	Autophagy-related lncRNAs.<br>Associated with neuroblastoma.	None
**BMF**<br>Bcl2 Modifying Factor	It has been shown to bind BCL2 proteins and function as an apoptotic activator. This protein is found to be sequestered to myosin V motors by its association with dynein light chain 2, which may be important for sensing intracellular damage and triggering apoptosis.<br>Downregulation is associated with progression of some cancer types.	Associated with follicular development (doi: 10.3390/ijms24010401)
