# Supplementary material for: CRISPR targeting of FOXL2 c.402C>G mutation reduces malignant phenotype in granulosa tumor cells and identifies anti‐tumoral compounds
Source: Mol Oncol. 2025 Jan 8;19(4):1092–116. doi: 10.1002/1878-0261.13799 (PMC11977662; doi:10.1002/1878-0261.13799)
Supplement: Supplementary file 15 — Table S7. Genes commonly de‐regulated in the transcriptomic and proteomic analyses. [file MOL2-19-1092-s010.pdf]

Supplementary Table 7. Genes commonly de-regulated in the transcriptomic and proteomic analyses. Up-regulated genes are in red and down-regulated genes in blue. The list of genes is ordered according to log2foldchange in the proteomic study.

Gene	log2Foldchange RNA	log2Foldchange PROT
RARRES2	8,84	5,97
FABP3	3,92	4,50
LMOD1	4,65	4,12
COL1A2	5,61	3,70
CSPG4	6,94	3,25
CAV1	4,08	3,24
ALDH1L2	1,64	3,14
TAGLN	6,08	3,04
PTN	1,77	2,89
TNC	2,94	2,89
PODXL	4,38	2,74
FLNC	4,64	2,50
CYSTM1	1,54	2,50
COL1A1	2,20	2,49
NCALD	2,31	2,47
CAV2	2,45	2,44
TPST2	1,75	2,32
ALDH1B1	2,60	2,23
LCP1	4,61	2,13
ENAM	3,90	2,07
SORBS1	4,86	2,07
GAS6	1,50	2,07
HSPA2	2,20	2,01
JPH2	3,63	1,95
ALCAM	1,60	1,92
FAS	1,62	1,82
MYOF	2,09	1,81
JAG1	2,51	1,72
COL4A1	1,82	1,72
GPC4	2,56	1,64
GPC1	1,53	1,64
CPA4	3,23	1,57
ITGA5	1,70	1,55
MYL9	3,41	1,52
NT5E	2,45	1,51
PTGIS	2,69	1,51
ASMTL	-1,78	-1,55
CADM1	-4,03	-1,62
VAT1L	-2,44	-1,74
SLC14A1	-4,18	-1,80
CYP11A1	-5,82	-1,89
LPXN	-1,66	-2,13
CALB2	-5,96	-2,44
ALDH1A3	-2,80	-2,44
