# Supplementary material for: CRISPR targeting of FOXL2 c.402C>G mutation reduces malignant phenotype in granulosa tumor cells and identifies anti‐tumoral compounds
Source: Mol Oncol. 2025 Jan 8;19(4):1092–116. doi: 10.1002/1878-0261.13799 (PMC11977662; doi:10.1002/1878-0261.13799)
Supplement: Supplementary file 16 — Table S8. List of top25 up (red) and down (blue) regulated proteins differentially expressed between PARENTAL and CRISPR clones. [file MOL2-19-1092-s007.pdf]

Supplementary Table 8. List of top25 up (red) and down (blue) regulated proteins differentially expressed between PARENTAL and CRISPR clones.

GENE	Function and relationship with cancer	Relation with granulosa cells and/or FOXL2	Common in Transcriptomic & Proteomic DEG
**RARRES2** Retinoic Acid Receptor Responder 2	Secreted chemotactic protein that initiates chemotaxis via the ChemR23 G protein-coupled seven-transmembrane domain ligand. This protein is involved in regulation of adipogenesis, energy metabolism, and inflammation. Related to ovarian cancer among others.	Regulates granulosa cells apoptosis during folliculogenesis by upregulating p53 and p21 (doi: 10.1002/mrd.23241). (doi: 10.1095/biolreprod.113.117044)	**Yes**
**FABP3** Fatty Acid Binding Protein 3	Participates in uptake, transport, and metabolism of long-chain fatty-acids in the cells. Tumor suppressor in breast cancer.	Participates in KGN cell proliferation and also increases the expression of aromatase CYP19 (doi: 10.1093/jmcb/mjaa044)	**Yes**
**LMOD1** Leiomodin-1	Required for proper contractility of visceral smooth muscle cells, it mediates nucleation of actin filaments. Deregulated in endometrial cancer (doi: 10.1038/s41598-020-66872-3).	Involved in ovulation in mouse ovary (doi: 10.1101/2023.08.21.554210).	**Yes (top-25)**
**COL1A2** Pro-alpha2 chain of type I collagen	Found in connective tissues, bone, cornea, dermis and tendon. Mutations and abnormal expression can cause instability of the extracelular matrix, increasing proliferation and invasion of tumoral cells.	Indicates oocyte quality, being upregulated in granulosa cells (doi: 10.1071/RD14452; 10.1016/j.rbmo.2021.05.018). FoxL2 (wild-type form) affects its transcription and expression, being increased when its knock-down and affecting follicular depletion during reproductive aging. Mutation FoxL2 C134W does not affect expression levels (doi: 10.1016/j.ydbio.2016.05.022)	**Yes**
**CSPG4** Chondroitin Sulfate Proteoglycan 4	It can mediate intracellular signalling downstream of growth factor receptor and integrin interactions, potentiating communication between the extracellular and intracellular compartments of the cell. Related to several tumor types.	Upregulated in TGFβ-induced GCTs, when compared with wild type granulosa cells (doi: 10.3390/cancers14092184)	**Yes (top-25)**
**CAV1** Caveolin 1	Main component of the caveolae plasma membranes. Negative regulator of RAS signaling. Links integrin to Ras-ERK signaling. It has been related to various cancer types, including head and neck squamous cell carcinoma, melanoma, and Ewing's sarcoma family tumors.	It plays a role in folliculogenesis and female reproduction (doi: 10.1093/HUMREP/DEY299). In bovine ovaries, CAV1 is expressed in granulosa and theca cells of the follicle and large and small cells of the corpus luteum (doi: 10.1002/MRD.20513).	**Yes (top-25)**
**ALDH1L2** Aldehyde Dehydrogenase 1 Family Member L2	This mitochondrial enzyme takes part in the folate metabolism (doi: 10.1074/jbc.M110.128843).	Related to cell cycle and apoptosis in primordial follicles (doi: 10.1093/toxsci/kfs137)	**Yes**

**TAGLN**<br>Transgelin	It is involved in calcium-independent smooth muscle contraction.<br>It acts as a tumor suppressor, and the loss of its expression is an early event in cell transformation and the development of some tumors, coinciding with cellular plasticity.	Upregulated in TGFβ-induced GCTs when compared with wild type granulosa cells (doi: 10.3390/cancers14092184).	**Yes (top-25)**
**ALDH2**<br>Aldehyde Dehydrogenase 2 or Mitochondrial	Mitochondrial isoform that participates in alcohol metabolism.	Deregulated in granulosa cells from PCOS patients (at protein level) (doi: 10.1177/11795514231206732)	No
**PTN**<br>Pleiotrophin	Secreted heparin-binding growth factor that participates in cell growth, migration, angiogenesis and tumorigenesis.	In the ovary is associated with stromal cell genes with COL1A1 (doi: 10.1038/s41421-022-00492-1). Under FOXL2 knock-down, this gene is upregulated in granulosa cells (doi: 10.1186/1471-213X-9-36)	**Yes**
**TNC**<br>Tenascin C	Component of the extracelullar matrix.<br>Contributes to elongation and migration of endothelial cells during angiogenesis in tumors. In ovarian tumors it appears to be overexpressed in the stroma and is also involved in invasion (doi: 10.1371/journal.pone.0136473).	None	**Yes**
**MCAM** Melanoma Cell Adhesion Molecule	Involved in glomerular filtration and vascular wound healing. Acts upstream of or within angiogenesis.<br>Biomarker of uveal melanoma.	Protein involved in neovascularization during the formation of corpus luteum in the human ovary, and defined as a FOXL2 target gene (doi: 10.1093/molehr/gag042; 10.7554/elife.04207)	No
**CD99**<br>T-Cell Surface Glycoprotein E2	Cell surface glycoprotein involved in leukocyte migration, transmembrane protein transport, rearrange cytoskeleton, T-cell adhesion and cell death.<br>Also plays an important role in the identification and diagnosis of different ovary tumors.	Granulosa cell tumors are positive for this cell marker, that is characteristic of sex cord-stromal tumors (SCSTs) (PMID: 9720506.) Granulosa cell tumor marker along with activin and inhibin-*α* (doi: 10.5858/2000-124-0563-IOISIA)	No
**PODXL**<br>Podocalyxin	Involved in cell adhesion and morphology in cancer progression by its interactions with actin-binding protein EZR, that increases activation of MAPK/PI3K pathway.	Related to ovarian cancer via activation by a miRNA (doi: 10.1007/s43032-020-00366-5)	**Yes**
**SDPR**<br>Serum Deprivation Response Protein (Cavin-2)	Protein increased in serum-starved cells and required for caveola formation.	None	No

**CD36** Platelet glycoprotein 4	Glycoprotein of the platelet surface, it serves as a receptor of thrombospondin in various cell lines	Overexpressed in granulosa cells of obese patients, causing malfunction of these cells and problems in fertility and ovarian function (doi: 10.1071/RD18292). Takes part in steroidogenesis and cholesterol pathways in granulosa cells by increasing its expression as a fatty acid transporter (doi: 10.1186/s40104-021-00660-5). Increased expression of CD36 reduces FOXL2 (doi: 10.1186/s12958-017-0276-z)	No
**CYSTM1** Cysteine-rich and transmembrane domain-containing protein 1	Implicated in immune system pathways. Biomarker of hepatocellular carcinoma	None	**Yes**
**FLNC** Filamin C	Related to myopathies and other muscular phenotypes.	Differentially expressed at transcriptomic level, when compared with *wild-type* FOXL2. It is associated with tumorigenesis due to its interaction with the extracellular matrix (doi: 10.1186/s12967-021-02754-0)	**Yes**
**COL1A1** Collagen Type I Alpha 1 Chain	Most abundant protein in the extracellular matrix.	Implicated in follicular phase, accumulating in cells due to its regulation via TGFβ signaling (doi: 10.1096/fj.202001377R).	No
**NCALD** Neurocalcin Delta	It is thought to be a regulator of G protein-coupled receptor signal transduction. Associated with ovarian cancer. Its lower expression in this case is related to chemotherapy resistance (doi: 10.1002/jcb.29670; 10.1186/s13048-020-00635-6)	None	**Yes**
**CAV2** Caveolin 2	Major component of the inner surface of caveolae, small invaginations of the plasma membrane, and is involved in essential cellular functions, including signal transduction, lipid metabolism, cellular growth control and apoptosis.<br>It plays a significant role in cancer progression and metastasis in various types of cancer, including head and neck squamous cell carcinoma (HNSCC), breast cancer, and pancreatic cancer.	None	**Yes (top-25)**
**TPST2** Tyrosylprotein Sulfotransferase 2	This type II integral membrane protein is found in the Golgi body catalyzes the O-sulfation of tyrosine residues within acidic regions of proteins.	None	**Yes**
**ALDH1B1** Aldehyde	Aldehyde dehydrogenase involved in involved in the metabolism of	Downregulated in cumulus cells (doi: 10.1093/biolre/ioab163)	**Yes**

dehydrogenases family of proteins	corticosteroids, biogenic amines, neurotransmitters, and lipid peroxidation. Contributes to proliferation of pancreatic cancer.		
**LCP1** Lymphocyte Cytosolic Protein 1	Actin-binding protein. It plays a role in the activation of T-cells in response to costimulation through TCR/CD3 and CD2 or CD28 and modulates the cell surface expression of IL2RA/CD25 and CD69. Associated with cancer.	None	**Yes**
**ENAM** Enamelin	Plays a role in the development of theet matrix. Downregulated in renal cancer tissues and also involved in inhibition of proliferation in clear cell renal carcinoma.	None	**Yes**
**ALDH1A3** Aldehyde dehydrogenase 1 family member A3	Catalyzes the NAD-dependent oxidation of aldehyde substrates. Promotes cancer cell growth and invasion inducing gene expression via retinoic acid.	Participates during steroidogenesis in the ovary (10.1016/j.jare.2023.06.002). ALDH1A3 enhances expression of ovarian genes in Sertoli cells, differentiating them into granulosa cells (doi: 10.1016/j.ydbio.2017.02.015)	**Yes**
**CALB2** Calbindin 2 **(Calretinin)**	Intracellular calcium-binding protein belonging to the troponin C superfamily. This protein plays a role in diverse cellular functions, including message targeting and intracellular calcium buffering. It plays a role in cancer progression and may serve as a potential biomarker or therapeutic target in certain cancer types.	Useful marker for GCTs (doi: 10.1309/GRH4-JWX6-J9J7-QQTA)	**Yes (top-25)**
**TXNIP** Thioredoxin-interacting protein	A thiol-oxidoreductase that is a major regulator of cellular redox signaling which protects cells from oxidative stress. Overexpression of this protein induces cell cycle arrest.	Downregulated in normal granulosa cells and up-regulated in PCOS granulosa patients, generating granulosa cell dysfunction by activation of NLRP3 inflammasome) (doi: 10.1016/j.mce.2022.111824)	No
**LPXN** Leupaxin	Focal-adhesion-associated adaptor-protein family member.	Upregulated with CD36 in PCOS patients with obesity (doi: 10.1071/RD18292)	**Yes**
**EEF1A2** Elongation factor 1 alpha 2	Promotes binding of aminoacyl-tRNA to the A-site of ribosomes in protein synthesis. Overexpressed in breast cancer, where is involved in migration and filopodia stimulation in a PI3K-Akt dependent manner.	Critical in the development of ovarian cancer and in Juvenile Granulosa cell tumors, causing cell migration (doi: 10.1016/j.ebiom.2015.03.002)	No
**SAT2** Serine acetyltransferase 2	Catalyzes the N-acetylation of the amino acid thialysine (S-(2-aminoethyl)-L-cysteine) and it is located in exosomes.	None	No

**CYP11A1** Cytochrome P450 Family 11 Subfamily A Member 1	Member of cytochrome P450 superfamily of enzymes, which catalyze many reactions involved in drug metabolism and synthesis of cholesterol, steroids and other lipids. This protein localizes to the mitochondrial inner membrane and catalyzes the conversion of cholesterol to pregnenolone, the first and rate-limiting step in the synthesis of the steroid hormones. It has been implicated in different cancers (breast, kidney, renal, squamous cell and skin).	It plays a crucial role in steroid hormone synthesis in granulosa cells. Increase expression in GCs undergoing luteinization in ovulation (doi: 10.1210/en.2016-1264. Epub 2016 Jul 18). No expression in granulosa cell tumors (doi: 10.1158/0008-5472.CAN-05-1024). FOXL2 represses the activity of the mouse Cyp11a1 promoter (doi: 10.1530/REP-11-0259).	**Yes (top-25)**
**HLA-DRB5; HLA-DRB3** Major Histocompatibility Complex Clas II DR Beta 3 and 5	HLA class II beta chain paralogues. They play a central role in the immune system by presenting peptides derived from extracellular proteins. Associated with gliomas with aggressive phenotypes.	None	No
**SLC14A1** Solute Carrier Family 14 Member 1	Membrane transporter that mediates urea transport in erythrocytes. Biomarker in some cancers. Associated with the progression of different tumors.	None	**Yes (top-25)**
**SERPINB2** Serpin Family B Member 2	Protein located on the external side of the plasma membrane, with endopeptidase activity, that promotes cell survival and it is associated with cytokine signaling. Overexpression inhibits apoptosis and promotes cell survival (doi: 10.1096/fj.202001377R).	Expression of this proteins depends on the stage of the follicular cycle. Highly upregulated in pregnant women (doi: 10.1016/j.theriogenology.2020.02.044). FOXL2 is defined as a positive regulator of this gene (doi: 10.3390/cancers11040499)	No
**LACC1** Laccase Domain Containing 1	Oxidoreductase that promotes fatty-acid oxidation, with concomitant inflammasome activation, mitochondrial and NADPH-oxidase-dependent reactive oxygen species production.	None	No
**VAT1L** Vesicle amine transport 1	Oxidoreductase and acetyltransferase activity. Involved in patient distant metastasis-free survival in breast cancer.	None	**Yes**
**RASGRF2** Ras Protein Specific Guanine Nucleotide Releasing Factor 2	Calcium-regulated nucleotide exchange factor that activates both RAS and RAS-related protein, RAC1.	Downregulated in the ovary of old mice (doi: 10.18632/aging.203150)	No
**GSTP1** Glutathione S-transferases	It plays an important role in detoxification by catalyzing the conjugation of many hydrophobic and electrophilic compounds with reduced glutathione. Related to susceptibility to cancer.	Hypermethylated in granulosa cell tumors, causing inactivation of this suppressor gene and increasing tumor progression (doi: 10.1158/1078-0432.CCR-04-0228)	No
**STK17B** Serine/Threonine Kinase 17B	This nuclear protein enables ATP binding activity and protein serine/threonine kinase activity. Involved in intracellular signal transduction; positive regulation of fibroblast apoptotic process; and protein phosphorylation.	None	No

	Highly expressed in various malignant tumors, including epithelial ovarian cancer (doi: 10.21037/atm-21-601)		
**TBCEL** Tubulin Folding Cofactor E like	Predicted to enable alpha-tubulin binding activity, to be involved in microtubule cytoskeleton organization, post-chaperonin tubulin folding pathway and tubulin complex assembly.	Upregulate in large follicles, include in cell cycle category (doi: 10.1186/1471-2164-15-24; 10.1101/2022.10.24.513438)	No
**TUBA1A; TUBA3E** Tubulins (Alpha a1 and e3)	Major components of the microtubules that take part in the formation of the cytoskeleton. Associated with tumor processes.	None	No
**HNMT** Histamine N-Methyltransferase	In charge of histamine degradation. The disregulation of its methylation involve this protein in certain cancers.	DEG in ovaries according to different environmental perturbations (doi: 10.1002/jez.b.22848)	No
**CADM1** Cell Adhesion Molecule 1	Involved in cell recognition, positive regulation of cytokine production, susceptibility to NK cell mediated cytotoxicity. Implicated in cervix, prostate and ovarian tumors, where it upregulates PI3K/Akt/mTOR signaling pathway, increasing cell proliferation and migration (doi: 10.1016/j.biopha.2019.109717)	None	Yes
**ACP1** Acid Phospatase 1	Hydrolyzes protein tyrosine phosphate to protein tyrosine and orthophosphate. Not related to cancer	None	No
**CAPG** Capping actin protein	Contributes to the control of actin-based motility in non-muscle cells.	None	No
**GLO1** Glyoxilase I	Linked to HLA. Participates in pyruvate metabolism and respiratory electron transport.	Involved in PCOS and decreased in old ovaries and ovulated oocytes, affecting their integrity (doi: 10.1016/j.fertnstert.2012.11.029)	No
**DPYD** Dihydropyrimidine Dehydrogenase	Catalyzes the reduction of uracil and thymine. Also involved in the degradation of the chemotherapeutic drug 5-fluorouracil	High expression in mural granulosa cells (doi: 10.1186/1471-2164-15-24)	No
**GALM** Galactose mutarotase	Cytoplasmic protein responsible for epimerization of hexose sugars such as glucose and galactose.	None	No
**ASMTL** Acetylserotonin O-Methyltransferase Like	Nucleoside triphosphate pyrophosphatase that hydrolyzes dTTP and UTP. May have a dual role in cell division arrest and in preventing the incorporation of modified nucleotides into cellular nucleic acids.	None	**Yes**
