# Supplementary material for: CRISPR targeting of FOXL2 c.402C>G mutation reduces malignant phenotype in granulosa tumor cells and identifies anti‐tumoral compounds
Source: Mol Oncol. 2025 Jan 8;19(4):1092–116. doi: 10.1002/1878-0261.13799 (PMC11977662; doi:10.1002/1878-0261.13799)
Supplement: Supplementary file 17 — Table S9. cMap first 100 ranked compounds that mimic the expression signature induced in KGN cells upon the elimination of FOXL2‐C134W mutation. [file MOL2-19-1092-s003.pdf]

Supplementary Table 9. cMap first 100 ranked compounds that mimic the expression signature induced in KGN cells upon the elimination of FOXL2-C134W mutation.

Rank	Score	Name	Description
1	99.93	azathioprine	Dehydrogenase inhibitor
2	99.86	SIB-1757	Glutamate receptor antagonist
3	99.72	**serdemetan***	MDM inhibitor
4	99.72	salvinorin-a	Opioid receptor agonist
5	99.68	paxilline	Potassium channel blocker
6	99.68	NSC-119889	Protein synthesis inhibitor
7	99.65	indole	aryl hydrocarbon receptor agonist
8	99.61	necrostatin-1	RIPK inhibitor
9	99.44	PF-3845	FAAH inhibitor
10	99.28	M-3M3FBS	phospholipase activator
11	99.22	**floxuridine***	DNA synthesis inhibitor
12	99.19	HNHA	HDAC inhibitor
13	99.19	KU-0060648	DNA dependent protein kinase inhibitor
14	99.08	APHA-compound-8	HDAC inhibitor
15	99.04	benzo(a)pyrene	Carcinogen
16	99.01	**everolimus***	MTOR inhibitor
17	99.01	**palbociclib***	CDK inhibitor
18	98.99	**olaparib***	PARP inhibitor
19	98.98	ISOX	HDAC inhibitor
20	98.98	BI-2536	PLK inhibitor
21	98.96	zebularine	DNA methyltransferase inhibitor
22	98.93	**tretinoin***	Retinoid receptor agonist
23	98.91	NCH-51	HDAC inhibitor
24	98.91	bumetanide	Solute carrier family member inhibitor
25	98.87	givinostat	HDAC inhibitor
26	98.87	THM-I-94	HDAC inhibitor
27	98.84	**belinostat***	HDAC inhibitor
28	98.84	**vorinostat***	HDAC inhibitor
29	98.81	papaverine	Phosphodiesterase inhibitor
30	98.8	HC-toxin	HDAC inhibitor
31	98.8	mycophenolic-acid	Dehydrogenase inhibitor
32	98.78	**panobinostat***	HDAC inhibitor
33	98.77	dacinostat	HDAC inhibitor
34	98.77	trichostatin-a	HDAC inhibitor
35	98.77	apicidin	HDAC inhibitor
36	98.77	scriptaid	HDAC inhibitor
37	98.77	XMD-892	MAP kinase inhibitor
38	98.7	XMD-1150	Leucine rich repeat kinase inhibitor
39	98.7	cyclazosin	Adrenergic receptor antagonist
40	98.63	alprazolam	Benzodiazepine receptor agonist
41	98.58	AG-14361	PARP inhibitor
42	98.52	entinostat	HDAC inhibitor
43	98.41	XMD-885	Leucine rich repeat kinase inhibitor
44	98.27	chromomycin-a3	DNA binding agent
45	98.26	methylene-blue	Guanylyl cyclase inhibitor
46	98.24	lofepramine	Norepinephrine reuptake inhibitor
47	98.22	montelukast	Leukotriene receptor antagonist
48	98.2	piceid	ICAM1 inhibitor
49	98.2	**alvocidib***	CDK inhibitor
50	98.17	ER-27319	Mediator release inhibitor
51	98.12	UB-165	Acetylcholine receptor agonist
52	98.03	pidorubicine	Topoisomerase inhibitor
53	98.03	mepacrine	Cytokine production inhibitor
54	97.99	PHA-793887	CDK inhibitor
55	97.99	hydrocotarnine	Opioid receptor antagonist
56	97.96	**topotecan***	Topoisomerase inhibitor
57	97.96	AT-7519	CDK inhibitor
58	97.96	pyroxamide	HDAC inhibitor
59	97.93	ketoconazole	Sterol demethylase inhibitor
60	97.92	cycloheximide	Protein synthesis inhibitor
61	97.9	lidoflazine	Calcium channel blocker
62	97.85	CFM-1571	Guanylate cyclase activator
63	97.82	Merck60	HDAC inhibitor
64	97.82	JNJ-7706621	CDK inhibitor
65	97.82	**daunorubicin***	RNA synthesis inhibitor
66	97.78	PIK-75	DNA protein kinase inhibitor
67	97.74	**camptothecin***	Topoisomerase inhibitor
68	97.74	triptolide	RNA polymerase inhibitor
69	97.69	prima-1-met	thioredoxin inhibitor
70	97.67	pirarubicin	Topoisomerase inhibitor
71	97.64	ZG-10	JNK inhibitor
72	97.64	WT-171	HDAC inhibitor
73	97.6	**mitoxantrone***	Topoisomerase inhibitor
74	97.57	5-iodotubercidin	Adenosine kinase inhibitor
75	97.57	KN-93	Calcium-calmodulin dependent protein kinase inhibitor
76	97.53	JWE-035	Aurora kinase inhibitor
77	97.51	naproxen	Cyclooxygenase inhibitor
78	97.46	**doxorubicin***	Topoisomerase inhibitor
79	97.43	menadione	Mitochondrial DNA polymerase inhibitor
80	97.38	**linsitinib***	IGF-1 inhibitor
81	97.32	anisomycin	DNA synthesis inhibitor
82	97.31	nornicotine	Acetylcholine receptor agonist
83	97.27	carvedilol	Adrenergic receptor antagonist
84	97.22	PAC-1	Caspase activator
85	97.21	WAY-629	Serotonin receptor agonist
86	97.15	HG-5-113-01	Protein kinase inhibitor
87	97.09	AG-490	EGFR inhibitor
88	97.04	clomifene	Estrogen receptor antagonist
89	97.03	bepridil	Calcium channel blocker
90	96.9	linifanib	PDGFR receptor inhibitor
91	96.9	PIK-90	PI3K inhibitor
92	96.86	ellipticine	Topoisomerase inhibitor
93	96.86	JNK-9L	JNK inhibitor
94	96.84	SN-38	Topoisomerase inhibitor
95	96.83	methyl-angolensate	Apoptosis inhibitor
96	96.77	PF-562271	Focal adhesion kinase inhibitor
97	96.73	AZD-6482	PI3K inhibitor
98	96.69	droxinostat	HDAC inhibitor
99	96.62	BMY-14802	Sigma receptor antagonist
100	96.58	cyclopamine	Smoothened receptor antagonist

* Drugs in common with Haltia et al. Gynecol Oncol. 2017
