# Supplementary material for: CRISPR targeting of FOXL2 c.402C>G mutation reduces malignant phenotype in granulosa tumor cells and identifies anti‐tumoral compounds
Source: Mol Oncol. 2025 Jan 8;19(4):1092–116. doi: 10.1002/1878-0261.13799 (PMC11977662; doi:10.1002/1878-0261.13799)
Supplement: Supplementary file 18 — Table S10. Types of compounds that mimic the expression signature induced in KGN cells upon the elimination of FOXL2‐C134W mutation. [file MOL2-19-1092-s017.pdf]

Supplementary Table 10. Types of compounds that mimic the expression signature induced in KGN cells upon the elimination of FOXL2-C134W mutation.

Compound (cp) description	# of cp
HDAC inhibitor	19
Topoisomerase inhibitor	8
CDK inhibitor	5
Acetylcholine receptor agonist	2
Adrenergic receptor antagonist	2
Calcium channel blocker	2
Cytokine production inhibitor	2
Dehydrogenase inhibitor	2
DNA synthesis inhibitor	2
JNK inhibitor	2
Leucine rich repeat kinase inhibitor	2
Opioid receptor antagonist	2
PARP inhibitor	2
PI3K inhibitor	2
Protein synthesis inhibitor	2
Adenosine kinase inhibitor	1
Apoptosis inhibitor	1
aryl hydrocarbon receptor agonist	1
Aurora kinase inhibitor	1
Benzodiazepine receptor agonist	1
Calcium-calmodulin dependent protein kinase inhibitor	1
Carcinogen	1
Caspase activator	1
DNA binding agent	1
DNA dependent protein kinase inhibitor	1
DNA methyltransferase inhibitor	1
DNA protein kinase inhibitor	1
EGFR inhibitor	1
Estrogen receptor antagonist	1
FAAH inhibitor	1

Compound (cp) description	# of cp
Focal adhesion kinase inhibitor	1
Glutamate receptor antagonist	1
Guanylate cyclase activator	1
Guanylyl cyclase inhibitor	1
ICAM1 inhibitor	1
IGF-1 inhibitor	1
Leukotriene receptor antagonist	1
MAP kinase inhibitor	1
MDM inhibitor	1
Mediator release inhibitor	1
Mitochondrial DNA polymerase inhibitor	1
MTOR inhibitor	1
Norepinephrine reuptake inhibitor	1
PDGFR receptor inhibitor	1
Phosphodiesterase inhibitor	1
phospholipase activator	1
PLK inhibitor	1
Potassium channel blocker	1
Protein kinase inhibitor	1
Retinoid receptor agonist	1
RIPK inhibitor	1
RNA polymerase inhibitor	1
RNA synthesis inhibitor	1
Serotonin receptor agonist	1
Sigma receptor antagonist	1
Smoothened receptor antagonist	1
Solute carrier family member inhibitor	1
Sterol demethylase inhibitor	1
thioredoxin inhibitor	1
